# Lipid Peroxidation and Depressed Mood in Community-Dwelling Older Men and Women

**DOI:** 10.1371/journal.pone.0065406

**Published:** 2013-06-11

**Authors:** Yuri Milaneschi, Matteo Cesari, Eleanor M. Simonsick, Nicole Vogelzangs, Alka M. Kanaya, Kristine Yaffe, Paola Patrignani, Andrea Metti, Stephen B. Kritchevsky, Marco Pahor, Luigi Ferrucci, Brenda W. J. H. Penninx, for the Health ABC study

**Affiliations:** 1 Department of Psychiatry and EMGO Institute for Health and Care Research, VU University Medical Center/GGZ inGeest, Amsterdam, The Netherlands; 2 Longitudinal Studies Section, Clinical Research Branch, National Institute on Aging, Baltimore, Maryland, United States of America; 3 Institut du Vieillissement, Université de Toulouse, Toulouse, France; 4 Department of Aging and Geriatric Research, University of Florida-Institute on Aging, Gainesville, Florida, United States of America; 5 Division of General Internal Medicine, University of California, San Francisco, San Francisco, California, United States of America; 6 Department of Psychiatry, University of California, San Francisco, San Francisco, California, United States of America; 7 Department of Neuroscience and Imaging and Center of Excellent on Aging, “G. d’Annunzio” University, Chieti, Italy; 8 Department of Epidemiology, Graduate School of Public Health, University of Pittsburgh, Pittsburgh, Pennsylvania, United States of America; 9 Sticht Center on Aging, Wake Forest University-School of Medicine, Winston-Salem, North Carolina, United States of America; Wayne State University, United States of America

## Abstract

It has been hypothesized that cellular damage caused by oxidative stress is associated with late-life depression but epidemiological evidence is limited. In the present study we evaluated the association between urinary 8-iso-prostaglandin F_2α_ (8-iso-PGF_2α_), a biomarker of lipid peroxidation, and depressed mood in a large sample of community-dwelling older adults. Participants were selected from the Health, Aging and Body Composition study, a community-based longitudinal study of older persons (aged 70–79 years). The present analyses was based on a subsample of 1027 men and 948 women free of mobility disability. Urinary concentration of 8-iso-PGF_2α_ was measured by radioimmunoassay methods and adjusted for urinary creatinine. Depressed mood was defined as a score greater than 5 on the 15-item Geriatric Depression Scale and/or use of antidepressant medications. Depressed mood was present in 3.0% of men and 5.5% of women. Depressed men presented higher urinary concentrations of 8-iso-PGF_2α_ than non-depressed men even after adjustment for multiple sociodemographic, lifestyle and health factors (*p = *0.03, Cohen’s d = 0.30). This association was not present in women (depressed status-by-sex interaction *p* = 0.04). Our study showed that oxidative damage may be linked to depression in older men from a large sample of the general population. Further studies are needed to explore whether the modulation of oxidative stress may break down the link between late-life depression and its deleterious health consequences.

## Introduction

Depression is highly prevalent in older persons and may complicate concurrent clinical conditions and increase the risk of disability and mortality [1]. The underlying pathophysiological mechanisms for this well-established association remain elusive.

Recent preclinical and clinical observations suggest that a potential role may be played by oxidative stress [2], in which production of highly reactive oxygen species (ROS) overwhelms antioxidant defenses in the cell and damage biomolecules (lipids, proteins, DNA) leading to mitochondrial dysfunction and cell death. Neurons are highly sensitive to oxidative stress due to their high metabolic rate and oxidative damage has been implicated in age-related neuronal decrement and neurodegenerative diseases [3]–[5]. Depression may be hypothesized to be a direct consequence of neurodegenerative processes. Indeed, brain imaging studies have linked depression with structural and functional alterations of limbic and cortical structures, particularly in the hippocampus [6]. Another hypothesis is that oxidative stress may impact on depression determinants. For instance, products of lipid peroxidation (such as isoprostanes) increase with age and are associated with multiple contributors to depression, such as cardiovascular disorders [7]; [8], obesity [9], stroke [10], diabetes [11], and inflammation [12]. Finally, oxidative stress may be also the consequence of depression, because depressed persons are more likely to engage in behaviors known to increase oxidative stress, such as smoking, excessive alcohol use, poor nutrition and sedentariness [13]; [14].

Research investigating the association between depression and oxidative damage is a relatively new area of study, and epidemiological evidence is limited. The few preliminary clinical studies have documented the association of depression with several biomarkers of protein (malondialdehyde [MDA]) [15]; [16], DNA (8-hydroxy-2′deoxyguanosine [8-OHdG]) [17]; [18], and lipid [19]–[21](8-iso-prostaglandin F_2α_ [8-iso-PGF_2α_]) oxidation. Nevertheless, existing studies characterized by small sample sizes have limited capacity to adequately take into account potential confounders and/or to perform secondary analyses in specific subgroups.

It has been proposed that biomarkers of lipid peroxidation may transduce the effect of oxidative stress associated with metabolic disturbances into specialized forms of cellular activation [13]. Previous epidemiological studies suggested that metabolic alterations (e.g. abdominal obesity, leptin dysregulation and inflammatory responses) were associated with late-life depression especially in men [22]–[24]. Moreover, data from the Health, Aging and Body Composition (Health ABC) study confirmed different immuno-metabolic profiles, due to difference in specific adipose depots, across gender/race groups [25]; [26].

Using data from Health ABC, we hypothesized that urinary concentrations of isoprostanes might be independently related with depressed mood in older persons. Thus, we examined the association between urinary excretion of 8-iso-PGF_2α_ and depressed mood in a large cohort of community-dwelling older persons taking important sociodemographic, health and lifestyle factors into account. Moreover, we explored whether this association differs according to gender and race.

## Methods

### Study Population

Data are from the Health, Aging and Body Composition (Health ABC) study, a cohort study consisting of 3075 initially well-functioning, community-dwelling persons aged 70 to 79 years at the baseline visit (1997–1998). Participants were identified from a random sample of white Medicare beneficiaries and all age-eligible community-dwelling black residents in designated areas surrounding Memphis, TN, and Pittsburgh, PA. Participants were considered eligible if they reported no difficulty walking one quarter of a mile, going up 10 steps without resting, and performing basic activities of daily living. Exclusion criteria included history of active treatment for cancer in the prior 3 years, plans to move out of the study area in the next 3 years, or participation in a randomized trial of a lifestyle intervention. Key components of Health ABC included an in-person interview and clinic-based examination, with evaluation of body composition, health conditions, and physical functioning. The primary measures were repeated at each annual clinic visit. The Institutional Review Boards at the University of Tennessee and at the University of Pittsburgh approved The Health ABC research protocol and the present study. Each participant signed an informed consent form. All clinical investigation has been conducted according to the principles expressed in the Declaration of Helsinki.

For the present analyses, we used cross-sectional data from the second visit (or first annual follow-up visit; 1998–1999) when urinary excretion of 8-iso-PGF_2α_ was ascertained as part of an ancillary research project examining the predictive value of this biomarker for the development of mobility disability [27]. Therefore, from the original Health ABC study sample, 32 participants who died during the first year, 648 who developed mobility disability before the analytical baseline and 111 who had missing or inadequate biological specimens were excluded. Excluded participants were older, more likely to be women and had higher prevalence of diabetes and cardiovascular disorders at the baseline visit [27]. Moreover, we excluded other 34 participants with missing data on depressive symptoms. Thus, the present analyses were based on the remaining 1975 participants.

### Depressed Mood

Depressed mood was operationally defined by the presence of relevant depressive symptoms and/or antidepressant use. Depressive symptoms were evaluated using the 15-item version of the Geriatric Depression Scale (GDS-15) [28]. Originally intended for screening depression in a population of non-demented elderly, it has shown adequate psychometric properties in community, primary care and hospital settings [29]. Items are summed and greater scores indicate a greater number of depressive symptoms. The GDS-15 does not contain items concerning somatic complaints. Presence of significant depressive symptoms was defined by the established cut-point of >5, which has a sensitivity of 90.9%, a specificity of 64.5%, a positive predictive value of 73.2% and a negative predictive value of 86.9% with respect to validated psychiatric diagnoses in elderly patients [30].

All medications regularly taken in the past 2 weeks were recorded according to the Iowa Drug Information System [31]. Antidepressant use (with depression/mood as self-reported indication) included monoamine-oxidase inhibitors (MAO-I), tricyclic antidepressants (TCA), selective serotonin reuptake inhibitors (SSRI), and other antidepressants.

### 8-iso-prostaglandin F_2α_

Measurement of the urinary excretion of 8-iso-PGF_2α_ has been demonstrated to be a reliable marker of lipid peroxidation in vivo [32]. Morning spot urine samples were collected from participants at their second annual clinical visit and stored at −70°C for subsequent analyses. Urinary concentrations of 8-iso-PGF_2α_ were measured by previously validated radioimmunoassay (RIA) methods [33] at the Laboratory of Clinical Pharmacology of Eicosanoids and Pharmacodynamic located in the Center of Excellence on Aging at the “Gabriele D’Annnunzio” University Foundation (Chieti, Italy). Measurements of urinary eicosanoid metabolites by RIA methods have been validated with different antisera and by comparison with gas chromatography-mass spectrometry [33]. The intra-assay and inter-assay coefficients of variation for the 8-iso-PGF_2α_ were 2.0% and 2.9% at the lowest level of standard (2 pg/ml), and 3.7% and 10.8% at the highest level of standard (250 pg/ml), respectively. Urinary levels of 8-iso-PGF_2α_ were adjusted for urinary creatinine (median: men 0.80 and women 0.56 mg/mL; p<.0001) to account for differences in renal excretory function. In the present analyses, concentration of 8-iso-PGF_2α_ was considered as a continuous measure as well as a dichotomous categorical variable (defined by the highest sex-specific quartiles; i.e., higher than 926.7 pg/mg-creatinine for men, and higher than 1,175.4 pg/mg-creatinine for women).

### Covariates

Covariates were a priori selected on the basis of previously reported associations with depression and oxidative stress. Sociodemographic variables included age, sex, race, educational attainment. Because a difference in median 8-iso-PGF_2α_ across study sites was found (Memphis 746.8 vs Pittsburgh 689.9 pg/mg-creatinine, Kruskall-Wallis *p* = 0.01), this covariate was retained in multivariate analyses. Alcohol consumption was assessed using a standardized questionnaire [34] and categorized as never, <1 drink/week, 1–7 drinks/week and >7 drinks/week. Body mass index (BMI) was calculated as kg/m^2^ and categorized as normal (BMI<25), overweight (25–29.99) and obese (BMI ≥30). Presence of cardiovascular diseases (CVD: heart failure, myocardial infarction, angina pectoris, coronary angioplasty or coronary artery bypass grafting), cerebrovascular diseases (stroke or transient ischemic attack) and diabetes were specifically ascertained using standardized algorithms [35], considering various information including self-report, medications, clinical findings, and medical claims data from the former Health Care Financing Administration. Each disease variable was then updated taking into account the new diagnoses and events reported by participants or determined by review of clinical documentation in the year prior to the second visit (the present study baseline). Since smoking status, comprehensive physical activity participation and cognitive function were not assessed at the second visit, these variables derive from data collected at the first Health ABC study visit. These dimensions are less likely to change during a 1-year period in this sample of well-functioning older persons [36]; [37]. Smoking status was coded as non-smoker/former/current smoker. Physical activity (in kcal/kg/week) was estimated through a specific questionnaire on the basis of to time/intensity spent on physical activities [38]. Cognitive status was evaluated with the Modified Mini Mental State Examination (3MS) [39].

### Statistical Analyses

Variables are presented as percentages, means (and standard deviations, SD) or median (and interquartile range, IQR). Univariate differences in sample characteristics were tested using chi-square or Kruskal-Wallis ANOVA, as appropriate. Differences in adjusted means of 8-iso-PGF_2α_ across depression status were examined using analyses of co-variance. For significant differences Cohen’s d [40] was calculated as a measure of effect size. In these analyses, concentrations of 8-iso-PGF_2α_ were log-transformed due to skewed distribution and mean values were presented back-transformed to facilitate interpretation. To investigate possible sex and race differences, depression status-by-sex and depression status-by-race interaction terms were entered in models including terms for depression status, age, sex, race, site, and education. Odds Ratios (ORs) and 95% Confidence Intervals (95%CI) of presenting depressed mood according to 8-iso-PGF_2α_ levels were estimated using logistic regression models. Analyses were adjusted for sociodemographic factors (age, race, education and site) and additionally adjusted for all the confounders (alcohol, smoking, 3MS, physical activity, BMI, cerebrovascular and cardiovascular disease and diabetes). Significance level was set at *p*<0.05. Because a statistically significant depression status-by-sex interaction was found, all results are shown for men and women separately. All analyses were performed using SAS (v. 9.2, SAS Institute, Inc., Cary, NC).

## Results

Main characteristics of the study sample are shown in Table 1. Participants’ mean age was 74.6 (SD 2.9) years; 48.0% of participants were women, and 35.7% black. The median level of 8-iso-PGF_2α_ was 721.3 (IQR 467.3–1052.3) pg/mg-creatinine. The overall prevalence of depressed mood was 4.2% (GDS-15 greater than 5∶1.4%; antidepressant use: 2.6%; both: 0.2%). Almost half of the sample (43.3%) had a GDS-15 score of 0. Participants on antidepressants, as compared to those drug-free, had significantly higher GDS-15 scores (1.9, SD 1.8 vs 1.1, SD 1.4; *p* = 0.001). Men, as compared to women, were older and less educated, had a lower cognitive function, and were more likely to drink alcohol, to smoke and to have diabetes and cardiovascular disorders. Moreover, men had lower concentrations of 8-iso-PGF_2α_ (median 633.4, IQR 420.1–929.4 vs 800.5, IQR 533.5–1178.9 pg/mg-creatinine; *p*<0.001) and a lower prevalence of depressed mood (3.0% vs 5.5%; *p* = 0.01).

**Table 1:** Main characteristics of the study population.

	Total sample	Men	Women	
Characteristics	(n = 1975)	(n = 1027)	(n = 948)	***p*** a
Age (*years*) *(mean±SD)*	74.6±2.9	74.9±2.9	74.6±2.8	0.02
Site *(Memphis)(%)*	49.7	49.0	50.5	0.50
Race (*Black*) (%)	35.7	32.5	39.0	0.003
Education *(%)*				<0.0001
less than high school	20.8	23.5	17.8	
high school	31.8	25.2	39.1	
postsecondary	47.4	51.4	43.2	
Alcohol intake *(%)*				<0.0001
never	47.5	41.8	54.4	
<1 drink/wk	21.2	20.3	22.1	
1–7 drink/wk	23.2	26.3	20	
>7 drink/wk	8.1	12.4	3.6	
Smoking status *(%)*				<0.0001
never smoker	44.7	31.7	58.8	
former smoker	46.9	9.1	7.7	
current smoker	8.4	59.2	33.5	
Physical activity *(Kcal/kg/week)(mean±SD)*	86.9±68.9	87.4±72.6	86.4±64.6	0.26
Modified Mini Mental State Examination *(mean±SD)*	90.9±8.0	90.0±8.4	91.8±7.5	<0.0001
Diabetes *(%)*	12.2	14.1	10.0	0.01
Cardiovascular disease (%)	15.7	21.5	9.4	<0.0001
Cerebrovascular disease *(%)*	6.0	5.9	6.0	0.95
Body mass index *(%)*				<0.0001
normal	34.5	30.4	38.9	
overweight	44.2	50.2	37.7	
obesity	21.3	19.4	23.4	
Depressed mood *(%)*	4.2	3.0	5.5	0.01
8-iso-PGF_2α_ *(pg/mg-creat.)(median and IQR)*	721.3 (467.3–1052.3)	633.4 (420.1–929.4)	800.5 (533.5–1178.9)	<0.0001

a from chi-square test or Kruskal-wallis ANOVA.

**Figure 1:**
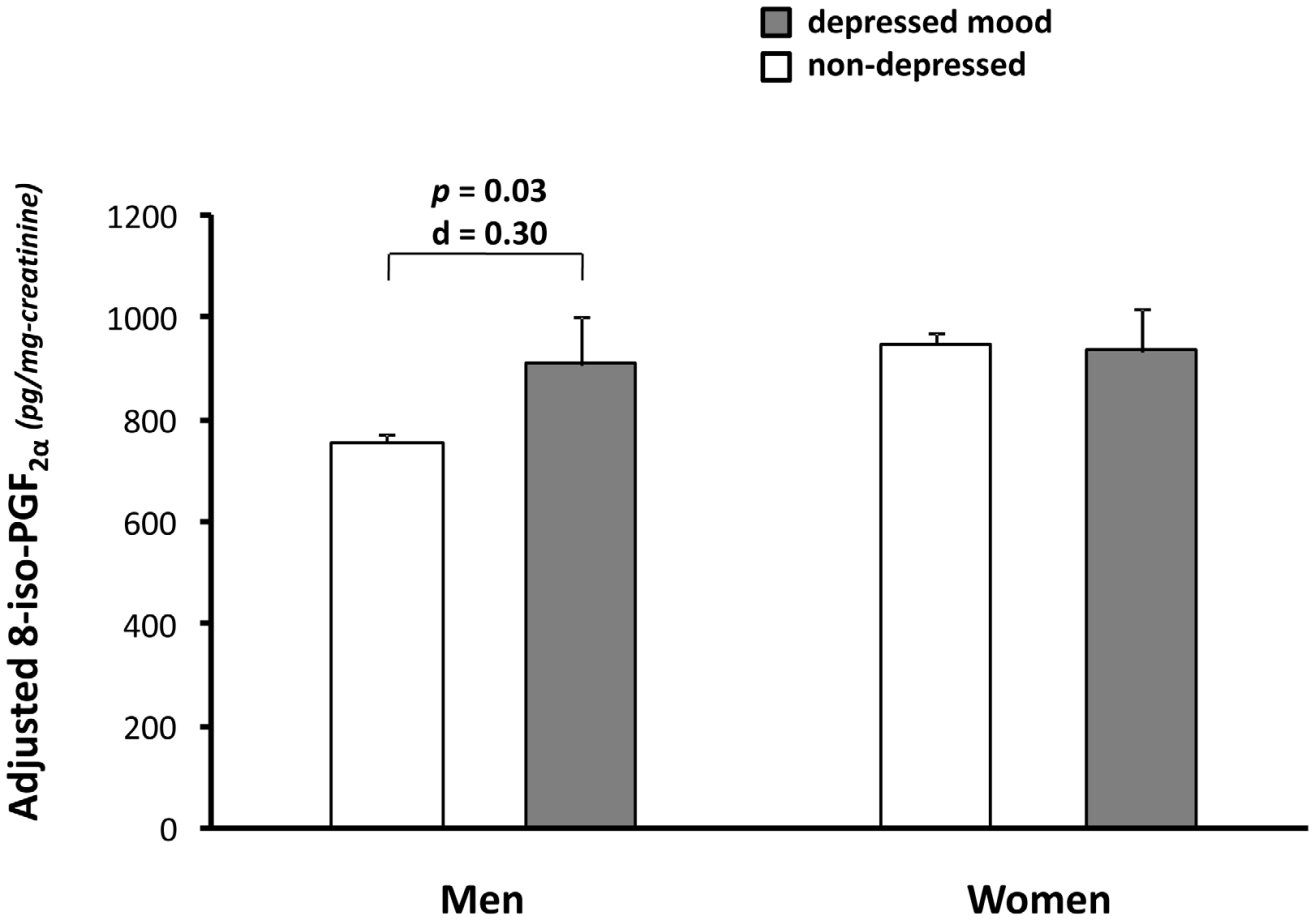
Adjusted means (and standard errors) of 8-iso-PGF_2α_ according to depression group in men and women. Values were adjusted for age, race, education, site, alcohol use, smoking, 3MSE, physical activity, BMI, cerebrovascular disease, CVD and diabetes. Statistical significance from analyses based on log-transformed 8-iso-PGF_2α_ due to skewed distribution.

After adjustment for sociodemographic factors, no statistically significant differences in 8-iso-PGF_2α_ levels were found between participants with depressed mood and those in the comparison group. However, a significant depression-by-sex interaction (*p* = 0.04) was found, whereas the depression-by-race interaction term was not statistically significant (*p* = 0.21). Figure 1 shows adjusted means (and standard errors) of 8-iso-PGF_2α_ according to depression status in men and women. Men with depressed mood had higher 8-iso-PGF_2α_ concentrations compared to non-depressed men, even after adjustment for multiple potential confounders (907.7, SE 95.2 vs 752.9, SE 16.2 pg/mg-creatinine, *p* = 0.03, Cohen’s d = 0.30). In contrast, no differences in 8-iso-PGF_2α_ were found in women with respect to presence of depressed mood (935.3, SE 82.2 vs 948.3 SE 19.7 pg/mg-creatinine, *p* = 0.51). In men, to test for a dose-response relationship we divided non-depressed participants into three groups according to GDS-15 scores selecting cut-off points to create subgroups with at least the same number of subjects as the depressed group. In a fully adjusted model of analysis of covariance we found a trend (*p = *0.08) in the adjusted means of 8-iso-PGF_2α_ across the different groups (depressed: 907.9 pg/mg-creatinine; GDS-15 score 4–5∶854.2 pg/mg-creatinine: GDS-15 score 1–3∶732.3 pg/mg-creatinine; GDS-15 score 0∶766.1 pg/mg-creatinine).

**Figure 2:**
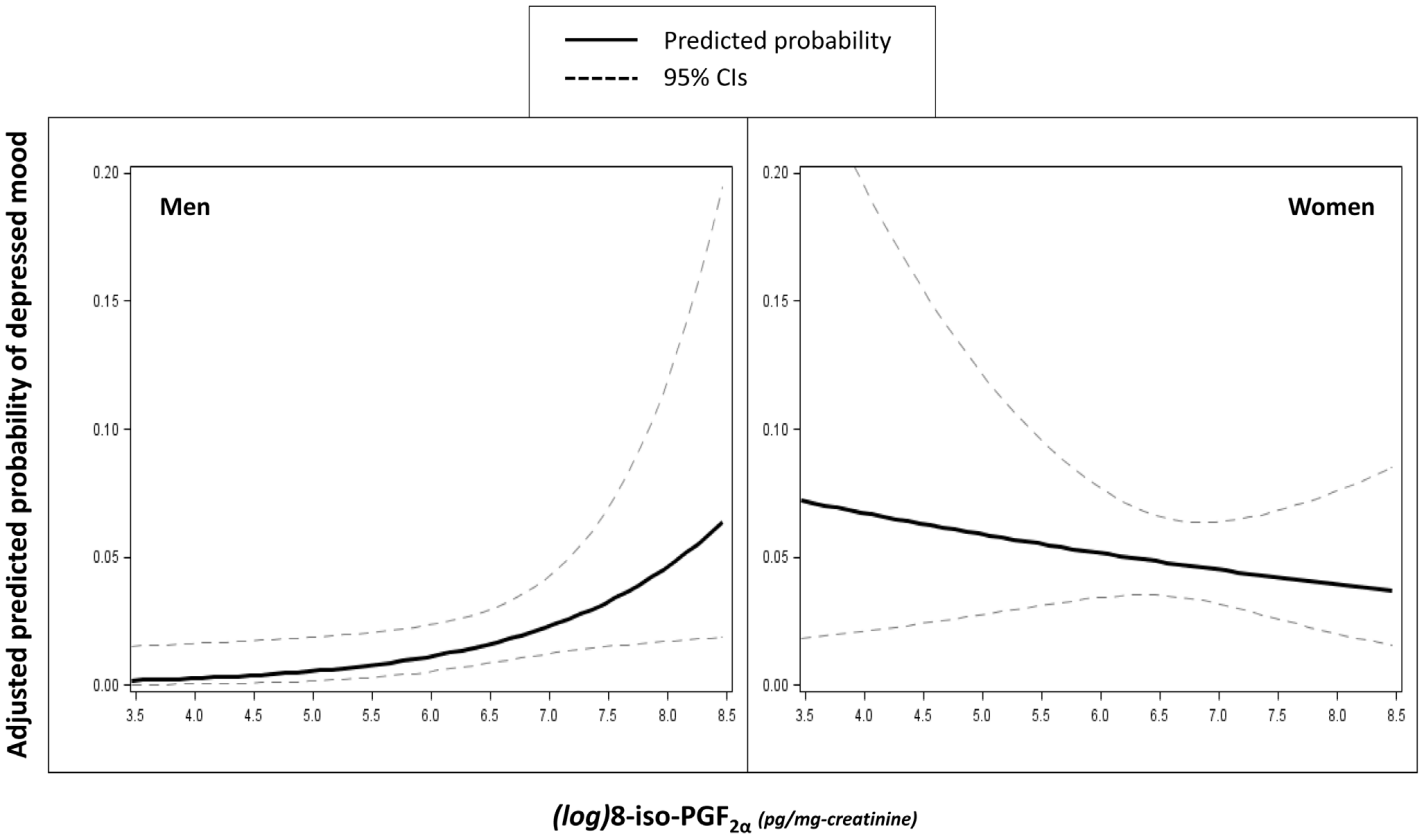
Plot of (log)8-iso-PGF_2α_ concentrations versus adjusted predicted probability of depressed mood according to sex. Predicted probability and 95% Confidence Intervals (95%CIs) are adjusted for age, race, education, site, alcohol use, smoking, 3MSE, physical activity, BMI, cerebrovascular disease, CVD and diabetes.

Table 2 shows the (adjusted) ORs of being depressed separately in men and women. After full adjustment, higher concentrations of 8-iso-PGF_2α_ were significantly associated with depressed mood in men (OR = 2.06, 95%CI = 1.09–3.89, *p* = 0.03), but not in women (OR = 0.87, 95%CI = 0.56–1.36, *p* = 0.53). Adjusted predicted probability of having depressed mood according to (log)8-iso-PGF_2α_ in men and women are plotted in Figure 2. Consistent results were obtained in analyses distinguishing participants with high (i.e. highest sex-specific quartile) versus normal (i.e. lower three quartiles) levels of 8-iso-PGF_2α_. Finally, similar results were obtained in sensitivity analyses performed after the exclusion of 107 participants with baseline cognitive impairment as indicated by a 3MS score below 80 [41] (data not shown).

**Table 2:** Odds ratios (and 95% confidence intervals) of depressed mood according to 8-iso-PGF_2α_ concentrations.

Association with Depressed Mood									
	Model 1			Model 2			Model 3		
	OR	95% CI	*p*	OR	95% CI	*p*	OR	95% CI	*p*
**Men** (prevalence depressed mood 3.0%)									
8-iso-PGF_2α_ *continuous* a	2.00	(1.12–3.56)	0.02	1.89	(1.05–3.42)	0.04	2.06	(1.09–3.89)	0.03
8-iso-PGF_2α_ *high (Q4) vs low (Q1-Q3)* b	2.55	(1.24–5.25)	0.01	2.40	(1.16–4.98)	0.02	2.61	(1.21–5.62)	0.01
**Women (**prevalence depressed mood 5.5%**)**									
8-iso-PGF_2α_ *continuous* a	0.93	(0.60–1.45)	0.76	0.87	(0.56–1.36)	0.54	0.87	(0.56–1.36)	0.53
8-iso-PGF_2α_ *high (Q4) vs ow (Q1–Q3)* b	0.80	(0.40–1.57)	0.51	0.75	(0.38–1.49)	0.41	0.73	(0.36–1.46)	0.37

a 8-iso-PGF_2α._ log-transformed.

b 8-iso-PGF_2α_. High levels (sex-specific highest quartile): >926.7 pg/mg-creatinine in men and >1,175.4 pg/mg-creatinine in women.

Abbreviations: OR, Odds Ratio; 95%CI, 95% Confidence interval.

Model 1: unadjusted.

Model 2: adjusted for age, race, education and site.

Model 3: additionally adjusted for alcohol use, smoking, Modified Mini Mental State Examination, physical activity, body mass index, cerebrovascular diseases, cardiovascular disease and diabetes.

## Discussion

The present study examined the association between a biomarker of oxidative damage and depressed mood in a large sample of community-dwelling older persons. After taking into account a large set of possible confounding factors, our analyses found the presence of a sex-specific relationship between urinary concentrations of 8-iso-PGF_2α_ and depressed mood. Depressed men showed higher levels of 8-iso-PGF_2α_ compared with non-depressed men. This association was of medium effect size, comparable to those found for inflammatory markers [42], brain-derived neurotrophic factor [43] and cortisol [44]. In contrast, no significant association was found in women.

In the present study only a small percentage of participants were classified as having depressed mood and almost half of the sample did not report any depressive symptoms. This may be due to the fact that the present analyses was based on a sub-sample from the Health ABC study [27] of non-disabled and well-functioning participants, therefore less likely to experience psychological distress. This is in contrast with the high prevalence of depressive syndromes commonly reported in older persons [1]. On the other hand, the selection of such persons decreases the probability that the association between depressed mood and oxidative damage may be biased by important confounding factors in late-life such as chronic diseases and disability.

The reasons for the lack of association in the present study between a measure of oxidative damage and depressed mood in women are unclear. Interestingly, we found that men, as compared to women, had lower levels of 8-iso-PGF_2α_ and less depressed mood. However, men were more likely to present characteristics associated with both oxidative stress and depression, such as smoking, cardiovascular diseases and diabetes. Gender differences in body composition may also have a role: men have more visceral fat, which is a major contributor to pathogenic immuno-metabolic responses linked to metabolic diseases and depression, while women have more subcutaneous fat, which has been associated to a more favorable immuno-metabolic profile [25]; [26]. It has been proposed indeed that F_2_-isoprostanes may transduce the effect of oxidative stress associated with complex metabolic disorders into specialized forms of cellular activation [13]. Interestingly, previous research showed that metabolic disturbances were associated with late-life depression especially in men. In the same Health ABC cohort, Vogelzangs et al. [22] reported that visceral fat was a risk factor for depression onset in older men. In a subsequent study [23] we showed that leptin dysregulation may represent an underlying mechanism for this relationship. In a recent study [24] based on a large sample including also patients with clinical diagnoses of depressive disorders, elevated inflammation was confirmed in depressed men, especially those with a late-onset depression. It is possible that in late-life depression metabolic-related oxidative injuries may have a larger role in men than in women. However, sex-specific differences in depression-related immuno-metabolic markers have not been found by all studies, and meta-analyses [42] including also younger samples showed a less clear moderation role for sex. Epidemiological studies on aging found gender-specific associations also with other biomarkers, such as plasma level of brain derived neurotrophic factor and personality traits linked to depression [45], although it is unknown whether this may have a relationship with oxidative stress. Moreover, in women depression may have a more complex etiology with other non-biological factors (e.g. social support, stressful life events) [46]; [47] playing stronger roles. Finally, the present study may have been underpowered to detect an association in women. Nevertheless, the above hypotheses are merely speculative and the sex-specificity of the present findings needs confirmation in future research.

Several mechanisms could explain the relationship between oxidative damage and depression. Neurons strongly depend on mitochondrial oxidative phosphorlation for energy, and unbalanced generation of ROS in the electrons transport chain may lead to oxidative damage, excitotoxicity and finally apoptosis. Because of its high oxidizable lipid content brain is highly affected by lipid peroxidation, which causes collapse of mitochondrial membranes [4]. The 8-iso-PGF_2α_ is indeed formed from peroxidation of arachidonic acid contained in phospholipids of cell membranes and its formation contribute to alterations in fluidity and integrity of membranes [13]; [48]. Furthermore, it has been demonstrated that isoprostanes have biological activities that may contribute to the progression of vascular damage inducing leukocyte adhesion and endothelial and platelet activation [49]. This is consistent with the well-established association between depression and cardiovascular diseases and the vascular hypothesis of depression suggesting that vascular damage in the brain predisposes to late-onset depression [50]. Oxidative stress might also trigger, trough activation of Nuclear Factor kB transcription activity, glia-mediated inflammatory responses linked to depression [4]; [42]. In this context, it is interesting to note the role of antioxidants in the reduction of lipid peroxidation by quenching free radicals. We recently showed [51] that low concentrations of serum carotenoids, powerful antioxidants embedded within the lipid layers that make up cell membranes, are associated with a higher risk of depression onset; this association was partially mediated by inflammation reduction. Moreover, it has been shown that stress-related adrenal glucorticoids (e.g. cortisol), whose alteration is considered an hallmark of some depressive syndromes, increase the vulnerability of hippocampal neurons to metabolic insult via impairment of antioxidant capacity [52]–[54]. Finally, oxidative damage may also be the consequence of depression, due to the fact that depression impacts health-related lifestyle such physical activity, diet, obesity and smoking all of which have been linked to oxidative stress [13]. However, in the present study adjusting for behavioral factors did not attenuate the association between 8-iso-PGF_2α_ and depressed mood. The effect of antidepressant treatment on oxidative stress markers has been examined in only a few studies with mixed results. Treatment with selective serotonin reuptake inhibitors has been shown to improve lipid peroxidation and to normalize initially disturbed antioxidant levels in depressed patients [55]. An in-vitro study [56] showed that tricyclic antidepressants increased generation of reactive oxygen species and decreased intracellular antioxidants; these effects were not produced by monoamine-oxidase inhibitors.

Our study has some important strengths, such as a large sample size, assessment of multiple confounders and the ability to test for sex and race differences. At the same time, some limitations are needed to be mentioned. First, since the study design is cross-sectional no inference about the directionality of the association can be made. Our analyses are based on single time-point assessments of the studied biomarker which may not be sufficient to adequately represent the participants’ true underlying metabolic state. Information on specific medications that are believed to lower urinary creatinine levels (e.g. Cephalosporins, Cimetidine, Cisplatin, Gentamicin, Trimethoprim) was not available. In addition, depression was not confirmed by a clinical diagnosis. In fact, depressed mood was operationally defined by GDS-15 scores and use of antidepressants. Given the very low prevalence of participants with depressed mood and the narrow variability of GDS-15 scores, we were unable to perform sensitivity analyses separating these two elements or using continuous GDS-15 scores. However, participants on antidepressants may represent the most severe case of depression (i.e., confounding by indication) and indeed in our sample those on antidepressants had significantly higher depressive symptoms.

Oxidative stress may represent one of the underlying mechanism linking late-life depression with negative health outcomes. A growing body of small clinical studies reported an association between oxidative damage markers and depression [15]–[21]. Our study adds to this growing literature by showing evidence for this association in a large sample of older persons from the general population. These findings underline the need for further studies in larger samples, well characterized in terms of psychiatric diagnoses and including measures of oxidative damage, in order to test whether oxidative stress is a cause, result or epiphenomenon of late-life depression and whether the modulation of oxidative stress may break down the link between depression and its deleterious health consequences.
